# Iduronate-2-Sulfatase-Regulated Dermatan Sulfate Levels Potentiate the Invasion of Breast Cancer Epithelia through Collagen Matrix

**DOI:** 10.3390/jcm8101562

**Published:** 2019-09-30

**Authors:** Vishal Singh, Keshav Kumar Jha, Jyothsna K. M, Rekha V. Kumar, Varun Raghunathan, Ramray Bhat

**Affiliations:** 1 Department of Molecular Reproduction Development and Genetics, Indian Institute of Science, Bangalore 560012, India; 2 Department of Electrical Communications and Engineering, Indian Institute of Science, Bangalore 560012 India; 3 Department of Pathology, Kidwai Memorial Institute of Oncology, Bangalore 560029, India

**Keywords:** dermatan sulfate, breast cancer, iduronate-2-sulfatase

## Abstract

Cancer epithelia show elevation in levels of sulfated proteoglycans including dermatan sulfates (DS). The effect of increased DS on cancer cell behavior is still unclear. We hypothesized that decreased expression of the enzyme Iduronate-2-sulfatase (IDS) can lead to increased DS levels, which would enhance the invasion of cancer cells. Breast cancer sections shows depleted IDS levels in tumor epithelia, when compared with adjacent untransformed breast tissues. IDS signals showed a progressive decrease in the non-transformed HMLE, transformed but non-invasive MCF-7 and transformed and invasive MDA-MB-231 cells, respectively, when cultured on Type 1 collagen scaffolds. DS levels measured by ELISA increased in an inverse-association with IDS levels. Knockdown of IDS in MCF-7 epithelia also increased the levels of DS. MCF-7 cells with depleted IDS expression, when imaged using two photon-excited fluorescence and second harmonic generation microscopy, exhibited a mesenchymal morphology with multiple cytoplasmic projections compared with epithelioid control cells, interacted with their surrounding matrix, and showed increased invasion through Type 1 collagen matrices. Both these traits were phenocopied when control MCF-7 cells were cultivated on Type 1 collagen gels polymerized in the presence of DS. In monolayer cultures, DS had no effect on MCF-7 migration. In the context of our demonstration that DS enhances the elastic modulus of Type 1 collagen gels, we propose that a decrease of IDS expression leads to accumulation within cancer epithelia of DS: the latter remodels the collagen around cancer cells leading to changes in cell shape and invasiveness through fibrillar matrix milieu.

## 1. Introduction

Breast cancer is the most common cancer occurring among women all over the world. Upon transformation, malignant epithelial cells breach their basement membrane and migrate through their surrounding stromal microenvironment [1]. The latter consists of resident cells such as fibroblasts and macrophages and a complex mixture of extracellular matrix proteins, which are primarily fibrillar, such as Type 1 collagen and elastin [2,3].

Migration may involve remodeling and degradation of collagen fibers by diffusible matrix metalloproteinases secreted by cancer epithelia or by active motility, which involves attachment to, and movement of cells along, matrix fibers with appropriate rearrangements of their microfilament cytoskeleton [4,5]. Such distinct mechanisms lead to diversity in the morphology of cancer cell migrations, from unicellular amoeboid or mesenchymal to collective multicellular modes [6]. In addition to negotiating their way through such collagen-rich matrices, cancer epithelia are able to effect changes in the pattern and arrangement of their surrounding collagen-rich matrices. This has been characterized historically by histopathologists as desmoplasia and consists of the alteration in the fibrillar patterns of existent, and freshly synthesized, collagen [7,8].

Rearrangement of collagen fibers by cancer cells may take place through distinct mechanisms. One such mechanism involves upregulation within cancer cells of lysyl oxidase (LOX), an enzyme that catalyzes cross-linking of collagen with elastin fibers [9,10,11]. Expression of LOX is a strong predictor for both migration and metastasis [10,12,13,14,15].

A second mechanism that cancer cells use to remodel their surrounding microenvironment is the expression of sulfated proteoglycans (PGs), proteins with one or more variable linear chains of repeating disaccharide units, known as glycosaminoglycans (GAGs) [16,17]. PGs are known to be elevated in, and are under active investigation as biomarkers for, cancer progression [18]. PGs have the ability to (re)constitute extracellular architecture through binding multiple proteins: both ligands and receptors that regulate cancer growth. They critically regulate the tumor cell motile phenotype by affecting their adhesive/migratory abilities and thus contribute to the metastatic cascade [18]. When cells are cultured in 3D matrix scaffolds, dermatan sulfate proteoglycans (DSPGs) are predominantly upregulated [19]. Dermatan sulfates, a type of GAG, have a unique disaccharide motif: N-Acetyl Galactosamine (GalNAc) and Iduronic Acid (IdoA), with potential sites for sulfation on either monosaccharide [20]. Decorin, one of the best studied DSPGs, binds to collagen through its protein core. The highly charged polysaccharide chains influence the material properties of the matrix by creating hydrogels through attraction of water through their high negative charge. Alterations in sulfation and proportion of GAGs such as chondroitin sulfate (CS) and dermatan sulfate (DS) have been reported in tumor transformation and progression [21], suggesting a possible mechanism by which CS/DS influences cancer progression. In contrast with CS, a potential role of DS in tumor progression is poorly understood.

DS GAGs get degraded in the lysosomes: the first step of degradation (hydrolysis of the C2-sulfate ester bond at the non-reducing end of 2-O-sulfo-α-L-iduronic acid residues) is mediated by the enzyme iduronate-2-sulfatase (IDS) through removal of sulfate groups from the glycan chain [22]. IDS belongs to the family of arylsulfatases, evolutionarily related enzymes that can hydrolyze sulfate esters of a variety of substrates such as sulfated GAGs, sulfo-lipids and -proteins, and steroids [23,24]. Mutations in the gene encoding IDS manifests as an X-linked lysosomal storage disease called mucopolysaccharidosis type II, also known as Hunter syndrome [25]. Deficiency in IDS leads to accumulation of DS in the tissues leading to an exaggeration of their ability to remodel Type 1 collagen [26].

In this manuscript, we begin by asking whether DS are elevated in breast cancer epithelia compared with untransformed breast cells, when cultivated in three-dimensional collagen scaffolds. Upon confirming the same, we show that this may occur through the downregulation of the DS degrading enzyme IDS, which in turn is elevated in non-cancerous epithelia located adjacently to tumor tissue wherein, its levels are low. This is consistent with the emerging evidence on the misregulation of several arylsulfatases upon malignant transformation of cells. Using a series of assays involving epifluorescence- and two-photon- and second-harmonic generation microscopy, we show how the deficiency of IDS in the cell line MCF-7 and the resultant accumulation of DS alters the interaction between Type 1 collagen and cancer cells promotes the invasion of the latter through stiffer fibrillar matrix environments.

## 2. Experimental Section

### 2.1. Antibodies and Reagents

The antibodies and reagents used in the study along with their source are as follows: goat polyclonal Iduronate 2-sulfatse (IDS) antibody (AF2449, R&D, Minneapolis, MN, USA) (used for immunocytochemistry (ICC) and immunohistochemistry), Mouse Anti-LAMP2 antibody (ab25631, Abcam, Cambridge, UK, used for ICC), Alcian blue 8GX (RM471) dye (HiMedia, Bangalore, India), Dulbecco’s modified Eagle medium (DMEM) (HiMedia, Bangalore, India), Fetal Bovine Serum (FBS) (Life Technologies, New York, NY, USA), penicillin-streptomycin (HiMedia, Bangalore, India), trypsin (HiMedia, Bangalore, India), paraformaldehyde (Merck, Bangalore, India), Triton X-100 (HiMedia, Bangalore, India), anti-goat antibody conjugated with Cy3 (Invitrogen, New York, NY, USA), anti-mouse antibody conjugated with Alexa fluor488 (Invitrogen, New York, NY, USA), DAPI (4′,6-Diamidine-2′-phenylindole dihydrochloride) (Invitrogen, New York, NY, USA), Phalloidin conjugated with Alexa fluor 488 and 660 (Invitrogen, New York, NY, USA), BSA (HiMedia, Bangalore, India), Type I collagen (Gibco, New York, NY, USA), Propidium iodide (HiMedia, Bangalore, India), TRIzol™ reagent (Invitrogen, New York, NY, USA), Turbofect (Thermo Fischer Scientific, Waltham, MA, USA), Dermatan sulfatase (Sigma, New York, NY, USA), Papain from papaya latex (P3125, Sigma, New York, NY, USA), 1,9-Dimethyl-Methylene Blue zinc chloride double salt (DMMB, 341088, Sigma, New York, NY, USA), and Iodoacetic acid (I4386, Sigma, New York, NY, USA).

### 2.2. Cell Culture

Human breast cancer cells MDA-MB-231 (transformed, triple negative, invasive) and MCF-7 (transformed non-invasive) were grown and maintained in DMEM supplemented with 10% FBS and 1X penicillin-streptomycin at 37 °C and 5% CO_2_ atmosphere. MDA-MB-231 culture media was also had Ham’s F-12 medium. Non-transformed HMLE cells were cultured in DMEM:F12 media (1:1) supplemented with insulin, EGF and hydrocortisone. 3D Type I collagen-rich extracellular matrix on-top and embedded cultures were prepared by seeding the trypsinized cells over and within a thin layer of 1 mg/mL Type 1 collagen. Type-1 collagen scaffolds were prepared by adding 8 volumes of acid-extracted unpolymerized Type 1 collagen (Gibco, USA) along with 1 volume of 10× DMEM and an appropriate volume of 0.1 N sodium hydroxide to bring the final concentration of polymerizing collagen to 1 mg/mL (pH: 7). The scaffolds were polymerized by incubating at 37 °C for 30 min in CO_2_ incubator. Subsequently, trypsinized cells were added on top or embedded in Type I collagen scaffolds and then cultured for 2 days in serum-free defined medium. 293FT cells (Invitrogen), used for lentivirus production, were grown and maintained in DMEM supplemented with 10% heat-inactivated FBS.

### 2.3. Lentiviral Vector Production and Transduction 

Lentiviral Iduronate 2 sulfatase (IDS) shRNA vectors (TRCN0000051543-7) for IDS knockdown and scrambled control shRNA were purchased from Sigma, USA. All lentiviral vectors were produced in 293FT cells by cotransfection of the IDS or scrambled control shRNA lentiviral vectors along with packaging plasmids (pLP1, pLP2, and VSVG, Addgene, Watertown, NY, USA) using Turbofect reagent (Thermo Fisher Scientific, Waltham, MA, USA) according to manufacturer instructions. The supernatant was collected 48 h and 72 h post-transfection and was centrifuged for 15 min at 3000× *g* and 4 °C to remove cell debris, and then passed through a 0.2-μm filter. Vector supernatants were concentrated 10–100 folds by using lenti-X concentrator (Takara Bio Inc., Kusatsu, Shiga, Japan) and centrifugation at 1500 g for 45 min at 4 °C. The pellet was dissolved in media and frozen at −80 °C. IDS shRNA stable cell lines were established by transducing MCF-7 cells with the purified virus, and stable pools of cells were selected with 1 μg/mL puromycin.

### 2.4. RT-qPCR

RNA from the cell lines (MDA-MB-231, MCF-7, HMLE, MCF-7 transduced with shRNA #1, #2 and scrambled) grown in 3D matrix of Type 1 collagen (1 mg/mL) for 48 h were isolated using TRIzol™ reagent (Thermo Fisher Scientific, USA) as per manufacturer’s protocol. Isolated RNA was quantified using UV-visible spectrophotometer (NanoDrop™). RNA (1μg) was reverse transcribed using Verso™ cDNA synthesis kit (Thermo Fisher Scientific AB-1453). All samples were processed at the same time and resulting cDNA was diluted 1:10. Real time PCR with SYBR green detection system (Thermo Fisher Scientific) was performed using StepOne Plus™ real-time PCR system (ABI) and IDS primers: Forward 5′-CGCGTTTCTTTCCTCACTGG-3′ and Reverse 5′-CCGACATGGTCACATAGCCA-3′ (Annealing temperature: 60 °C). Appropriate no-RT and no template controls were included in each biological repeat. 18S rRNA was used as internal control gene for normalization.

### 2.5. Alcian Blue Staining of Breast Tissues and Cell Lines

Adjacent breast and tumor sections were de-paraffinized and hydrated with distilled water. Sections were then incubated in Alcian Blue (1%, pH 1.0) for 1 h, washed in running tap water and then rinsed in distilled water. Finally, the sections were dehydrated through graded alcohols, cleared, mounted and photographs were captured using Olympus Ix81 microscope equipped with a digital camera. Pixel quantification of the Alcian blue stain was done using Image J software. For this, the captured images were first converted to grayscale, inverted and then equal area was selected in the luminal epithelia of healthy breast, and cancer epithelia for the comparison of Alcian blue pixels. Similarly, HMLE, MCF-7, and MCF-7 cells transduced with lentiviral IDS shRNAs and scrambled control vectors and MDA-MB-231 were grown on Type I collagen (300 μg/mL) for 48 h, fixed with 4% formaldehyde, washed with PBS and stained with Alcian blue (1%, pH 1.0) overnight. Thereafter, cells were washed with PBS, investigated for autofluorescence from the dye (green in color) through laser scanning confocal microscopy using a LSM 880 with Airyscan (Carl Zeiss, Oberkochen, Germany) microscope and analyzed by ZEN 2.1 (blue edition, Carl Zeiss, Oberkochen, Germany) software. Thereafter, pixel quantification of the autofluorescence from Alcian blue stain was done in the captured images using Image J 1.52a (National Institutes of Health, Rockville, USA) software. Scatter plots of pixel intensity in the case of tissue and cell lines were plotted using GraphPad 5.0 (GraphPad Software Inc, San Diego, USA) software.

### 2.6. Immunocytochemistry

About 5 × 10^3^ cells (MDA-MB-231, MCF-7, HMLE, MCF-7 cells transduced with IDS shRNA #1 or shRNA #2 or scrambled shRNA) were seeded on the top of Type I collagen scaffolds (1 mg/mL) per well of 8-well chamber slide (Eppendorf, New York, NY, USA). After 48 h, the cells were washed with phosphate-buffered saline (PBS) twice, fixed with 4% paraformaldehyde (for 20 min) and permeabilized using 0.1% Triton-X100 (15 min) at room temperature (RT). The cells were then washed 3 times with PBS and incubated with the blocking solution (1% BSA in PBS) for 1 h at RT. The cells were then incubated with the primary antibodies, anti-IDS at 1 μg/mL and anti-LAMP2 at 1/100 dilution, for overnight at 4 °C, washed 3 times with PBS plus 0.1% Tween-20 thereafter for 15 min, and incubated with secondary antibodies (Alexa561-tagged anti-goat and Alex488-tagged anti-mouse) and phalloidin conjugated with Alexa 633 (each at 1/500 dilution) for 1 h at RT. Thereafter, cells were washed with PBS three times and incubated with DAPI (1 μg/mL) and fluorophore-conjugated phalloidin for 10 min at RT, followed by rinsing with PBS. The images of 3D cultures were obtained by laser scanning confocal microscopy using a LSM 880 with Airyscan (Carl Zeiss, Oberkochen, Germany) microscope and analyzed by ZEN 2.1 (blue edition, Carl Zeiss, Oberkochen, Germany) software.

### 2.7. Immunohistochemistry

The 5-μm-thick tissue sections were made from paraffin embedded blocks of breast cancer patients from Kidwai Memorial Institute of Oncology with informed consent of the patients. The slides were first deparaffinized and were then subjected to antigen retrieval using citrate buffer. Thereafter, the tissue sections were immunostained with goat anti-IDS antibody (1 μg/mL) overnight at 4 °C. The sections were then incubated with Alexa 561-tagged anti goat secondary antibody at 1/500 dilution for an hour at RT. Thereafter, cells were washed with PBS three times and incubated with DAPI (1 μg/mL) for 10 min at RT, followed by rinsing with PBS and mounted with glycerol. All sections were photographed using epifluorescence microscope (Olympus IX81, Center Valley, USA) and identical exposure times.

### 2.8. In Vitro Invasion Assay

The membrane on the top chamber (12-well insert; pore size 8 μm, HiMedia, Bangalore, India) was coated with a mixture of 200 μg/mL of Type I collagen and allowed to polymerize in absence or presence of 50 μg/mL of dermatan sulfate (Sigma, New York, NY, USA), overnight in CO_2_ incubator. In the inserts where Type I collagen was polymerized in absence of dermatan sulfate (DS), 3 × 10^4^ of MCF-7 cells transduced with IDS shRNA or scrambled shRNA were seeded on the top chamber in medium without serum and medium with serum was placed in lower chamber as a chemoattractant. Similarly, in the inserts where Type 1 collagen was polymerized in presence of DS, 3 × 10^4^ of wild type MCF-7 cells were seeded. Appropriate controls were also maintained in each experiment. The cells were incubated for 48 h and non-invasive cells were removed by cotton swab. The invasive cells were fixed, stained for DAPI and analyzed using epifluorescence microscope (Olympus IX81, Center Valley, USA). The number of invaded cells on each whole membrane was counted. 

### 2.9. ELISA for DS Estimation

The in vitro quantitative determination of DS concentrations in lysates of HMLE, MDA-MB-231, MCF-7 cells, MCF-7 cells transduced with IDS shRNA or scrambled shRNA, cultured in 3D matrix of Type I collagen (1 mg/mL), was carried out using ELISA kit (Elabscience, Houston, TX, USA). The micro ELISA plate provided in this kit was pre-coated with an antibody specific to Human DS and its estimation was done according to manufactures recommendation. Briefly, a standard solution of DS ranging from 20 ng/mL to 0.31 ng/mL was prepared. Next, 100 μL of standard or cell lysate (prepared by digesting 3D collagen culture using papain and hence representing the microenvironmental sGAG consisting of both intracellular and matrix-sequestered sGAGs) were added to each well of ELISA plate and incubated for 90 min at 37 °C. Thereafter, liquid was removed and 100 μL of biotinylated detection antibody were added to each well which was incubated for 1 h at 37 °C. Then, liquid was aspirated, and washing was done 3 times with wash buffer. Next, 100 μL of HRP conjugate were added and incubated for 30 min at 37 °C. After aspirating and washing 5 times with wash buffer, 90 μL of substrate reagent was added and incubation was done for 15 min at 37 °C. After this, 50 μL of stop solution was added and the color that developed was read at 450 nm immediately (Tecan infinite M200 Pro™, Mannedorf, Switzerland). The four-parameter logistic (4PL) curve model was used to analyze and quantify the DS levels. The DS levels were normalized to total protein as quantified using Bradford assay.

### 2.10. Scratch Assay

The effect of DS on migration capacities of MCF-7 cells was assessed by in vitro wound healing or scratch assay. Approximately 2 × 10^4^ cells were seeded per well of an 8-well chamber slide and grown to a confluence of 80–90% at 37 °C in an atmosphere of 5% CO_2_:95% air. A wound was created by scraping the cells with a sterile 200 μL pipette tip in the middle of the culture well. After removing cellular debris with sterile PBS, cells were incubated with 50 μg/mL of DS for 48 h. An untreated control was also maintained. Thereafter, cells were stained with DAPI and wound closure photographs were captured using Olympus Ix81 epifluorescence microscope equipped with a digital camera and analyzed using Image J software. 

### 2.11. Dimethylmethylene Blue (DMMB) Assay

Sulfated GAGs can be measured directly by use of a metachromatic dye, 1,9-Dimethylmethylene blue (DMMB, 341088, Sigma, New York, NY, USA). The GAG-dye complex results in an absorption spectrum shift that can be measured at between 515 and 530 nm, which is directly proportional to the amount of sulfated GAGs. HMLE, MDA-MB-231 and MCF-7 cells cultured in 3D matrix of Type I collagen (1 mg/mL) for 48 h were digested with papain (300 μg/mL) at 60 °C for 3 h. Thereafter, iodoacetic acid was added to a final concentration of 10mM. A standard curve of chondroitin 4 sulfate ranging 0–10 μg/mL was also prepared. Then, 50 μL of lysate or standard were added to 50 μL of DMMB dye in a microplate well and the plate was read at 525 nm in a microplate reader (Tecan infinite M200 Pro™).

### 2.12. Experimental Setup for Two Photon Microscopy and Image Analysis

A mode-locked fiber laser (Coherent Fidelity HP) with operational wavelength 1040 nm, pulse width of 140 fs and repetition rate of 80 MHz was used as the fundamental excitation to acquire second harmonic generation (SHG) and two photon emission fluorescence (TPEF) images of the samples. The incident beam was scanned using a galvo-scanner (GVS001, Thorlabs, Newton, MA, USA) with the beam focused on the sample using a 60× water immersion objective (NA 1.2, Olympus, Shinjuku, Japan). The SHG emission signal from the collagen and TPEF images from the cell of the same field of view were collected separately using photomultiplier tube (R3896, Hamamatsu, Japan) in epi-detection using two different filter sets with wavelength range of 520 nm ± 20 nm and 605 nm ± 55 nm, respectively. The incident power at the focus for cell and collagen was 2.5 mW and 7.5 mW, respectively. The optical resolution of the multi-photon imaging was estimated to be ~600 nm. Four different field of views of 50 × 50 microns size of cell and collagen surrounding the cell along and two different field of views of the collagen far away from the cell were imaged.

### 2.13. Atomic Force Microscopy (AFM)

For AFM measurements, Type 1 collagen polymerized in the absence or presence of 50 µg/mL was freshly prepared and incubated in PBS until acquisition. The apparent modulus of elasticity of the cells was measured using an Atomic Force Microscope (XE Bio from Park Systems, Suwon, Korea). We used a V-shaped cantilever with a spherical bead of diameter 5.2 mm made of silicon dioxide attached to its bottom (AppNano HYDRA6V-200NG-TL; AppNano, Mountain View, CA, USA). The stiffness of the cantilever was measured using a thermal tuning method available with the AFM and was found to be 0.041 N/m. The relation between the deformation of the cantilever and the voltage on the photodetector (A-B sensitivity) was calibrated by indenting the cantilever on the petri dish. The calibration was done whenever the laser position on the cantilever was adjusted. We used a cantilever speed of 0.8 mm/s while approaching as well as retracting from the cell. The point was designated as the modulus of the cell. For obtaining the elastic modulus and the point of contact from the F-d curves, we used the Hertzian contact model as follows. First, the approach region of the F-d curve when the cantilever is not in contact with the cell is identified, and the force in this region is corrected to zero. In this region, the F-d curve is linear and almost flat. A straight line is fitted to this region, and this line is subtracted from the F-d curve to correct for the baseline force. The elastic modulus and contact point are now obtained from the baseline-corrected F-d curve by fitting a Hertzian contact model for the region between 0.2 and 2 nN.

### 2.14. Statistical Analysis

Data are presented as mean ± standard error of mean. Unpaired *t*-test was used to compare between groups and one-way ANOVA with Tukey’s post-hoc test was used for comparison of more than two groups. *p* < 0.05 was considered significant. All statistical analyses and graphs were plotted using GraphPad Prism 5.0 (GraphPad Software Inc., San Diego, CA, USA).

## 3. Results

### 3.1. Sulfated Proteoglycans Are Elevated in Breast Cancer Epithelia in Vivo and in Culture 

**Figure 1:**
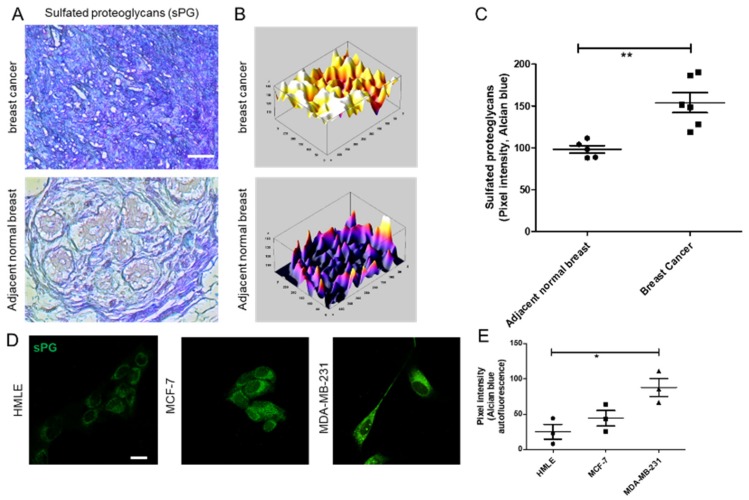
Sulfated proteoglycans are elevated in breast cancer epithelia in vivo and in culture. (**A**) Breast cancer (top) and patient-matched adjacent non-transformed (bottom) breast tissue sections were stained with Alcian blue dye (blue), which detects sulfated proteoglycans (sPGs) at pH 1. Scale bar = 50 µm. (**B**) 3D profile plots representing sPGs levels in tissue sections of breast cancer (top) and adjacent normal breast (bottom) with bright colored peaks showing higher staining for Alcian blue in breast cancer tissues (blue-low; white-high). (**C**) Scatter plot showing pixel intensities of Alcian blue staining in breast cancer and adjacent normal breast tissue sections (*n* = 5). (**D**) Confocal micrographs of Alcian blue autofluorescence (green signal) in stained immortalized breast epithelial cells HMLE (left), non-invasive malignant MCF-7 (middle) and the triple negative invasive MDA-MB-231 cells cultured on top of 1 mg/mL Type 1 collagen gels. Scale bar = 20 µm. (**E**) Scatter plot showing pixel intensities of Alcian blue autofluorescence in stained HMLE, MCF-7 and MDA-MB-231 cells cultured on top of 1 mg/mL Type 1 collagen gels (lines in graphs represents mean ± SE of three independent experiments). Significance was measured using one-way ANOVA (* *p* < 0.05) and student’s *t* test (** *p* < 0.01).

To assess the levels of sulfated proteoglycans (sPGs) between normal and malignant breast epithelia, we stained five sets of sections of breast cancer and patient-matched adjacent tissue with Alcian blue, a dye that specifically binds to sulfated mucopolysaccharides at pH = 1.0 [27]. The tumor cells were found to stain to a greater extent for sPGs than the cells that constituted the normal acinar architectures in the adjacent areas (Figure 1A–C; blue represents sPGs). To confirm that the sPGs were being secreted by cancer cells, the untransformed HMLE cells, non-invasive MCF-7 cells and the triple-negative invasive MDA-MB-231 cells were cultured on top of Type I collagen matrix scaffolds, fixed and stained with Alcian blue. When compared to HMLE, MCF-7 and to a greater extent MDA-MB-231 cells showed significantly higher signals for sPGs (Figure 1D, E; green fluorescence represents sPGs (see Figure S1), results with another dye staining sGAGs, DMMB, are shown in Figure S2, and a plot of individual cell autofluorescent signals is shown in Figure S3). Alteration in material properties of the tumor microenvironment has been shown to profoundly affect cancer invasion [28]; moreover, among sPGs, DS is increasingly shown to alter the fibrillar properties of collagenous microenvironments and is also preferentially upregulated within 3D cultures [19]. Therefore, we next asked whether the enzymes regulating the levels of DS were responsible for the elevated levels of sPGs.

### 3.2. Decreased IDS Expression and High DS Levels in Cancer Epithelia 

**Figure 2:**
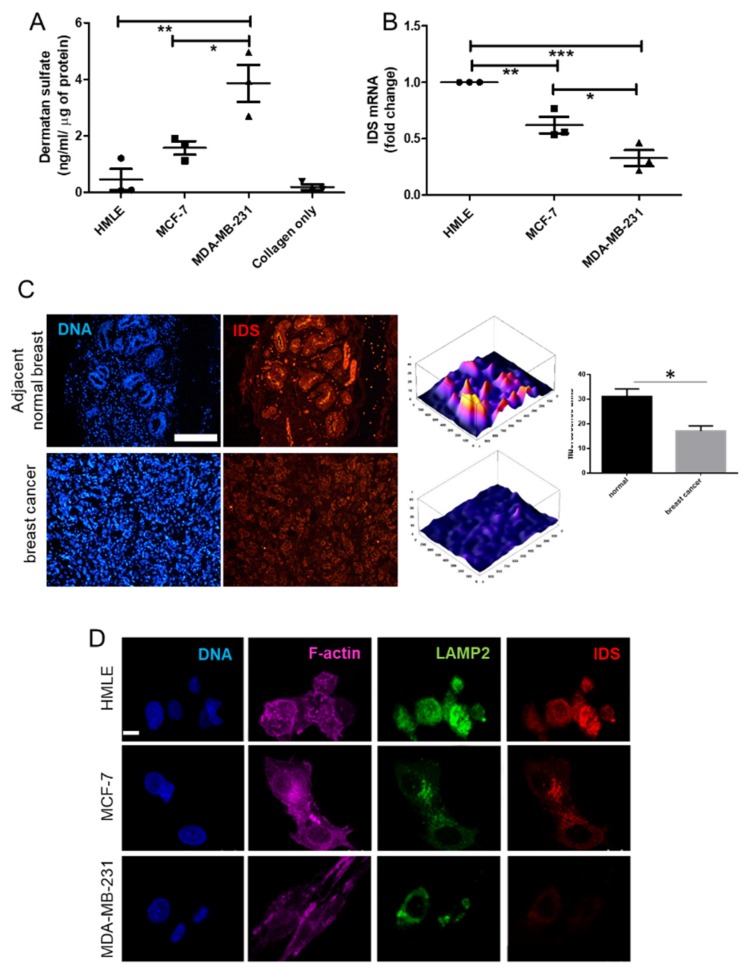
Levels of iduronate-2 sulfatase (IDS) are decreased in cancer epithelia in vivo and in culture. (**A**) Scatter plot of dermatan sulfate (DS) levels normalized to total protein measured using ELISA in HMLE, MCF-7 and MDA-MB-231 cells cultured in 3D Type 1 collagen scaffolds. (**B**) Scatter plot of IDS mRNA levels in HMLE, MCF-7 and MDA-MB-231 cells cultured in 3D Type 1 collagen scaffolds, as determined by real time PCR with 18S rRNA as internal control. (**C**) (left) Epifluorescence micrographs of matched normal breast sections (top) and breast cancer tissues (bottom) stained for DNA (using DAPI; blue), and IDS (using antibody; red), scale = 200 μm. (middle) 3D profile plots of IDS levels in normal (top) and cancer (bottom) sections with bright colored peaks showing higher staining for IDS in nontransformed cells. (right) Bar graph showing a statistically significant decrease in IDS levels in breast cancer tissues compared with adjacent breast epithelial cells (50 cells, 5 fields, 2 sample sets). (**D**) Confocal micrographs of HMLE, MCF-7 and MDA-MB-231 cells cultured on top of Type 1 collagen scaffolds and stained for DNA (with DAPI; blue), F-actin (with phalloidin; pink), acidic compartment (with antibody against LAMP2; green), and for IDS (antibody; red), scale = 20 µm. Significance was measured using one-way ANOVA and student’s *t* test (* *p* < 0.05, ** *p* < 0.01, *** *p* < 0.001).

To ascertain whether DS were being secreted by cancer cells to a greater extent than untransformed cells, ELISA was performed on the lysates of 3D Type I collagen cultures of HMLE, MCF-7 and MDA-MB-231 cells. DS levels, normalized to total proteins were highest in MDA-MB-231, followed by MCF-7 and lowest in HMLE lysates (Figure 2A).

IDS mediates the first step of the degradation of DS, through hydrolysis of sulfate ester bonds at the non-reducing end of 2-O-sulfo-α-l-iduronic acid [25]. Hypothesizing that the accumulation of DS could be explained by a decrease in expression of IDS (based on reported decrease in mRNA levels in human breast tissues with increased stage of cancer progression, specific histopathological types, and increased lymph node metastasis in The Cancer Genome Atlas, Figure S4), we assessed the transcript levels of IDS in breast cells cultured in Type 1 collagen scaffolds, using quantitative real-time PCR (qRTPCR): mRNA levels were highest in HMLE, followed by MCF-7 and lowest in MDA-MB-231 (Figure 2B). The relatively higher levels of IDS have been earlier reported in the context of non-invasive MCF-7 and T47D breast cancer cell lines [29]. We then performed immunohisto- and cyto-chemical analysis to assess IDS protein levels in breast cancer epithelia in cancer tissues and 3D collagen cultures of cell lines, respectively. We found that IDS protein was highly expressed in non-cancerous acinar epithelia with very sparse staining in cancer cells within the sections (Figure 2C). In fixed and stained 3D cell cultures, HMLE showed highest levels of IDS, followed by MCF-7, with sparse staining in case of MDA-MB-231. Given the known canonical localization of IDS in lysosomes, we stained the acidic compartments of cells using LAMP-2. In both HMLE and MCF-7, the IDS signals were colocalized with LAMP-2 (Figure 2D; no-primary antibody control for IDS staining is shown in Figure S5). We then asked whether depletion of IDS within the non-invasive MCF-7 cells would affect their morphological phenotype.

### 3.3. IDS Knockdown Leads to Higher DS Levels

**Figure 3:**
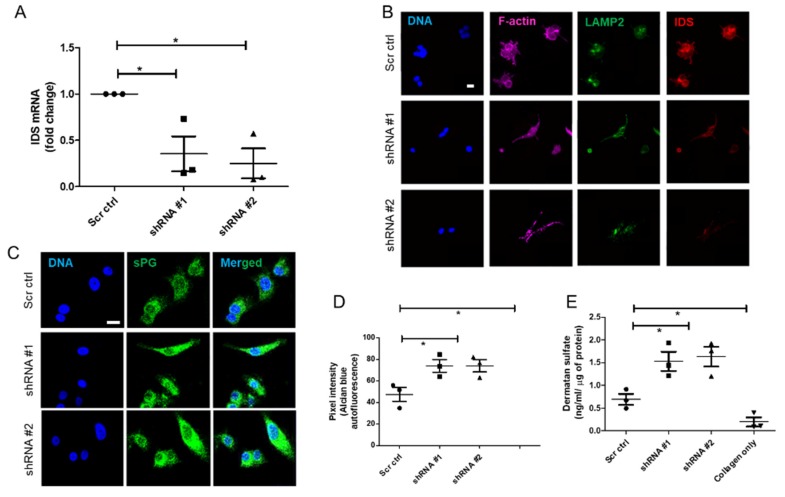
Decrease in IDS levels increases DS levels and alters the shape of MCF-7 cells. (**A**) Graphical representation showing a downregulation of IDS mRNA levels in MCF-7 cells upon lentiviral transduction of 2 shRNA clones, compared with scrambled control shRNA transduction, using qRT-PCR. 18S rRNA was used as an internal control. (**B**) Confocal micrographs of MCF-7 cells with scrambled- and IDS-specific shRNA transduction, stained for DNA (using DAPI, blue), F-actin (using phalloidin, pink), acidic compartment (using anti-LAMP-2 antibody, green) and IDS (using anti-IDS antibody, red). Depletion of IDS is accompanied with change in shape of MCF-7 cells from polyhedral to a spindle-like morphology, scale = 10 µm. (**C**) Confocal micrographs of MCF-7 cells with scrambled- and IDS-specific shRNA transduction, stained for DNA (using DAPI, blue), and sulfated proteoglycans (using Alcian Blue, green), scale = 20 µm. (**D**) Scatter plot representation of the pixel intensities of autofluorescent signals from Alcian Blue staining from 3C. (**E**) Scatter plot representation depicting dermatan sulfate (DS) levels in control and IDS knockdown MCF-7 cells when cultured in 3D Type 1 collagen scaffolds, analyzed using ELISA. Levels are represented as scatter plots (mean ± SE of three independent experiments). Significance was measured using one-way ANOVA (* *p* < 0.05).

Stable repression of IDS expression was carried out using cognate shRNA (two distinct clones), through lentiviral transduction in MCF-7 cells. IDS knockdown was assessed using qRTPCR, which showed that considerably lower mRNA levels in MCF-7 cells transduced for both shRNA clones compared with control MCF-7 transduced with a scrambled shRNA in 3D Type I collagen cell cultures (Figure 3A). Using immunocytochemistry, we also found a decrease in IDS protein levels in MCF-7 cells transduced with either of the IDS shRNA clones, as compared to scrambled shRNA controls, when the cells were cultured in Type I collagen (Figure 3B). It is pertinent to point out that we also noticed a difference in cell shape concomitant with IDS perturbation: compared with the typical polygonal shape of MCF-7 cells cultured on top of Type 1 collagen, IDS-depleted cells had a more spindle-like appearance typical of mesenchymal cells. We then asked whether a decrease in IDS levels increases sPG levels. IDS-depleted MCF-7 cells grown on Type 1 collagen, fixed and stained with Alcian blue, showed significantly higher levels of sPGs, compared to scrambled shRNA transduced MCF-7 cells (Figure 3C,D). We first assessed whether IDS knockdown also resulted in increased DS levels within MCF-7 cells. This was confirmed using ELISA, in lysates of IDS-depleted MCF-7 cells compared with scrambled shRNA controls when the cells were grown in 3D Type 1 collagen scaffolds (Figure 3E).

### 3.4. IDS Downregulation Increases Invasion of Mesenchymal MCF-7 Cells through Collagen Matrices

**Figure 4:**
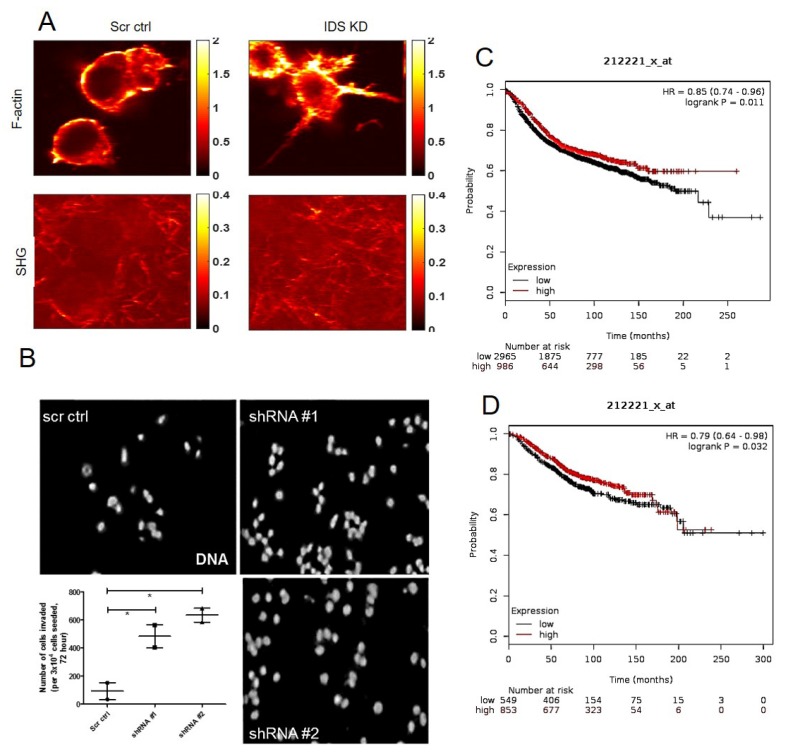
Low levels of IDS increase invasion of MCF-7 and correlate with poorer prognosis of breast cancer patients. (**A**) Two photon micrographs of MCF-7 control cells (left) and with IDS knockdown (right) showing phalloidin staining of actin cytoskeleton (top) and second harmonic generation signals (bottom), scale = 10 µm. (**B**) Epifluorescence micrographs of the invasion of MCF-7 cells stained for DNA (using DAPI), lentivirally transduced with scrambled shRNA and 2 shRNA clones against IDS through transwells coated with Type 1 collagen. DAPI was used as indicator of invaded cells. Graphical representation of the number of invaded cells shown in 4B. (**C**,**D**) Kaplan–Meier plots of risk-free survival and overall survival, respectively, reveal a significant correlation between higher IDS expression and better survival (lines in graphs represent mean ± SE of 2–3 independent experiments). Significance was measured using one-way ANOVA (* *p* < 0.05).

We next examined the effect of IDS depletion on the shape of MCF-7 cells cultured in 3D collagen scaffold in greater detail using two-photon excitation fluorescence (TPEF) microscopy accompanied by second harmonic generation (SHG) imaging with the help of F-actin stained cells. IDS-depleted cells had numerous cytoplasmic projections from their surface which was found to be seen rarely in the case of the polygonal control MCF-7 cells. In addition, collagen fibers, well organized around control MCF-7, were found to be spatially coincident with cells upon IDS knockdown indicative of greater cell-matrix interaction (Figure 4A). To further probe such interactive behavior, we cultured cells within transwells coated with Type 1 collagen and assessed their propensity for invasion. IDS shRNA-transduced MCF-7 cells showed significantly greater invasion compared to scrambled shRNA-transduced MCF-7 cells (Figure 4B). Kaplan–Meier plots for risk-free and overall survival showed that higher IDS levels correlated with better prognosis among patients whose expression levels were curated within GEO, EGA and TCGA databases [30] (Figure 4C,D).

### 3.5. DS-Type 1 Collagen Scaffolds Increase Invasion of Mesenchymal MCF-7 Cells

**Figure 5:**
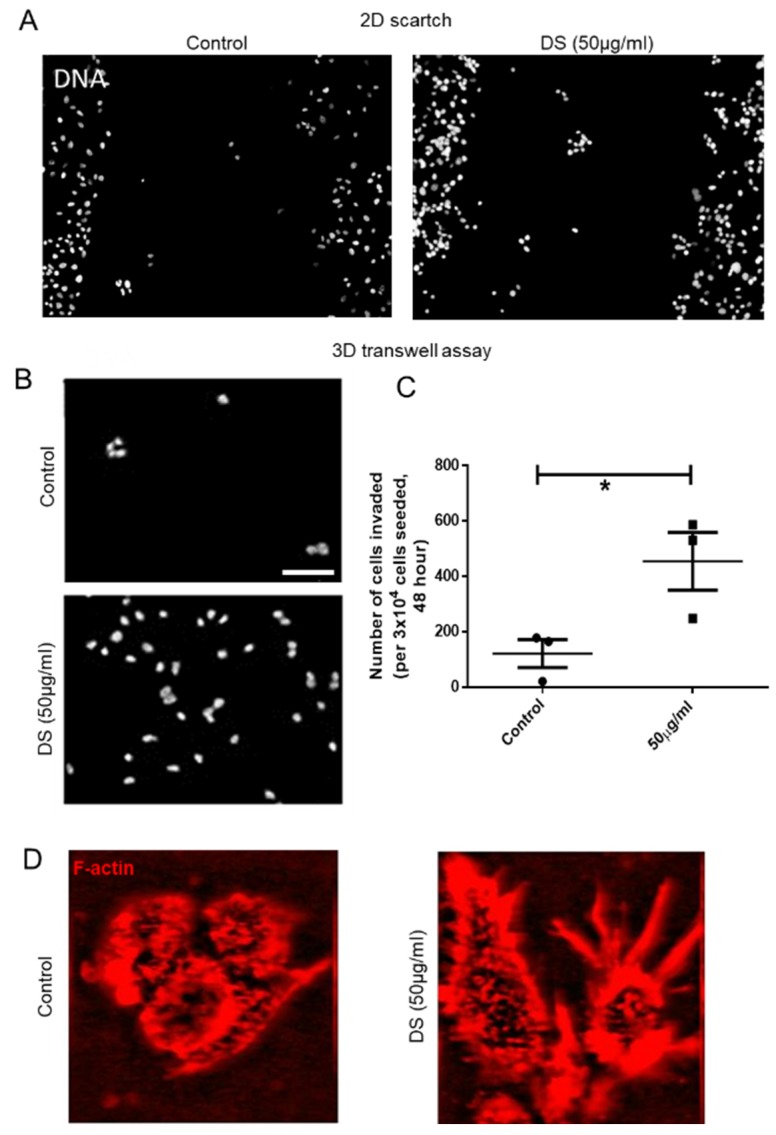
Increase in DS levels phenocopies IDS depletion and increases MCF-7 invasion. (**A**) Epifluorescence micrographs showing lack of migration of MCF-7 within scratches made in monolayers (left, control; right, upon treatment with 50 µg/mL Dermatan Sulfate (DS)). The cells were visualized by staining DNA using DAPI. (**B**) Epifluorescence micrographs showing invasion of MCF-7 through Type 1 collagen-coated transwells (top, control; bottom, transwells coated with Type 1 collagen scaffold polymerized in the presence of 50 µg/mL DS). The cells were visualized by using DNA stain DAPI. (**C**) Scatter plot showing the number of invaded cells in 5B. (**D**) Two-photon micrographs of MCF-7 cells cultured in 3D Type 1 collagen scaffold (left) and in 3D Type 1 collagen scaffolds polymerized in the presence of 50 µg/mL DS (right) showing F-actin staining (using phalloidin, red) (lines in graphs represent mean ± SE of three independent experiments). Significance was measured using Student’s *t* test (* *p* < 0.05).

To assess whether the increased invasiveness of mesenchymal MCF-7 was a direct result of elevated DS, or an indirect effect of the latter on Type 1 collagen polymerization, scratch assays were performed wherein the ability of MCF-7 cells to fill a scratch were assessed in the presence or absence of 50 μg/mL of DS added to the medium. In both cases, MCF-7 cells were unable to fill the scratch (Figure 5A). On the other hand, in transwells coated with Type 1 collagen, which was polymerized in the presence of 50 μg/mL of DS, MCF-7 cells showed greater invasion compared with controls (Figure 5B,C). Assessed with TPEF microscopic imaging of F-actin, MCF-7 cells grown on top of DS-spiked Type 1 collagen scaffolds also exhibited a more mesenchymal phenotype with several cytoplasmic protrusions (Figure 5D).

## 4. Discussion

The last decade has seen unprecedented advances in our understanding of the mechanical cues exchanged between cancer epithelia and their matrix microenvironments [31]. Malignantly transformed cells mount a complex response on their surrounding matrix glycoproteins that are part of the basement membranes and the surrounding collagen-rich stroma. The response consists of direct degradation of protein and glycan molecules through upregulation of proteases (such as MMPs) and glycosidases (such as heparanases) [32,33]. Degradation may also be mediated through the activation of tissue-resident fibroblasts which can remodel the matrix within the cancer niche [34,35]. Distinct from degradation, cancer cells and activated fibroblasts also secrete unique matrix proteins into their surrounding milieu. The proteins are referred to as the cancer matrisome and may serve as unique signatures for diagnosis and prognosis of cancer [36,37]. The cancer matrisome differs from untransformed cell secreted matrices not just in their proteomic composition but also in their glycan content. The latter in turn alters the patterning and linkages between matrix proteins resulting in specific mechanical changes in the cancer microenvironment [38]. What is the nature of such glycans and how are they upregulated?

To address these questions, we examined the effect of a specific GAG: dermatan sulfate in tumor environments. The rationale for choosing DS was twofold: Firstly, proteoglycans bearing DS such as decorin alter collagen fiber patterns. Secondly, unlike heparan sulfates, the role of DS still remains ill-understood in the context of carcinomatosis. In consonance with our hypothesis, the expression levels of DS-degrading enzyme IDS were depleted in all histological types of cancers when examined in the TCGA database and observed to be decreased in invasive malignant epithelia compared with non-invasive or untransformed breast epithelia. This was concomitant with an increase in DS levels within the extracellular milieu. The decrease in levels of IDS in non-invasive MCF-7 cells not only increased their invasion, but also the shape of these cells on collagen matrices, polygonal in control cells, underwent a change to a more mesenchymal type with cytoplasmic protrusions. The incorporation of GAGs has been elegantly shown to alter cell-ECM interactions and induce the acquisition of mesenchymal phenotype [39]. The transformation in cellular phenotype is therefore, likely the consequence of the upregulation in DS levels and its alteration in polymerization pattern of Type 1 collagen. This is because MCF-7 cells cultured in Type 1 collagen that was polymerized in the presence of DS, also showed an increase in cell invasion and mesenchymal cell shape. To examine the effect of DS on collagen polymerization, we examined the control and IDS-depleted MCF-7 cells grown on Type 1 collagen scaffolds using SHG microscopy. We detected a clear separation between control MCF-7 cells and surrounding collagen fibers. On the other hand, SHG signals of fibers were intermingled with cells that had depletion of IDS, suggesting greater interaction between cells and extracellular matrix (ECM). Our findings suggest that the presence of DS in Type I collagen changes the rheological properties, making it more permissive to better cell-matrix adhesion and invasion. In line with this, preliminary observations using atomic force microscopy indicate an increase in stiffness of DS-spiked Type 1 collagen matrix when compared with unspiked control matrix (Figure S6).

Our observations suggest that DS secreted by transformed cells can alter the mechanical properties and polymeric arrangement of surrounding collagen fibers leading to enhanced cell matrix interaction and mesenchymal migration. Our findings raise several important questions. Does the alteration in collagen polymer patterning as a result of increased DS further feedback on IDS expression? Important observations by workers in the field show that fibroblasts grown in 3D embedded in collagen make more DS than HS, with the latter bound to the collagen fibers [19]. However, it is not known if this increase in DS is through the downregulation of IDS.

Second, in what way does DS alter the patterns of Type 1 collagen in order to bring about an increase in invasiveness of cancer epithelia? DS consists of iduronic acid, which, being inherently more flexible than glucuronic acid, can bind to its cognate binding partners much more strongly [40,41]. DS is known to bind to growth factors [42]. Therefore, high levels of DS in the collagenous milieu may also enhance the sequestration of growth factors in the vicinity of cells leading to better availability of these ligands for cell proliferation and migration.

Finally, we asked how the presence of DS in the collagen allows the cells to stretch and change its shape. It is possible that DS, while increasing stiffness of Type 1 collagen scaffold, also alters the interfacial tension between the cells and matrices allowing for a greater optimal contact between the cell and their surrounding fibers. The increase in sulfation of GAGs in concurrence with epithelial to mesenchymal transition (EMT) has previously been reported. Maupin and coworkers, using a diverse set of pancreatic cancer cell lines and Alcian blue, found that mesenchymal cancer cells show increased sulfation. Inducing EMT in Panc-1 mesenchymal-like cell line with TGFβ also led to a significant increase in overall levels of sulfation by altering the expression of sulfotransferases [43]. Our study is complimentary to these efforts in demonstrating that not only the increase in sulfotransferases, but also a depletion of sulfatases may lead to EMT. Future experimentations will be devoted to elucidation of the mechanochemical effect of DS on collagen fibrillogenesis as well as investigating the effects of DS on expression of cell adhesion molecules and cytoskeletal elements that determine cell shape change. 

## 5. Conclusions

We conclude by proposing that the progression towards invasiveness of transformed breast epithelia is associated with an appropriate decrease in desulfation of dermatan sulfate (DS) proteoglycans leading to their accumulation within their surrounding stromal collagen-rich matrix. The accumulated DS modifies Type 1 collagen and potentiates the invasion of breast cancer cells. Therefore, our findings potentially open a new window for therapeutic targeting of dermatan sulfates in order to decrease the burden of cancer metastasis and invasion.

## Supplementary Materials

The following are available online at https://www.mdpi.com/2077-0383/8/10/1562/s1, Figure S1: Bright field (left) and laser confocal (right) micrographs of the same field of Alcian blue-stained MCF-7 cells cultured on top of 1 mg/mL Type 1 collagen gels. Images show that the levels of green autofluorescence signals correspond to the intensity of Alcian blue staining in the brightfield. Scale bar = 50 μm, Figure S2: Scatter plot of DMMB assay depicting levels of sulfated glycosaminoglycans (GAGs) in HMLE, MCF-7 and MDA-MB-231 cells grown in 3D Type-I collagen scaffolds. (Data is mean ± SE of three independent experiments). Significance was measured using one-way ANOVA (* *p* < 0.05, ** *p* < 0.01), Figure S3: Scatter plot showing pixel intensities of Alcian blue autofluorescence in stained HMLE, MCF-7 and MDA-MB-231 cells cultured on top of 1 mg/mL Type 1 collagen gels. Each spot represents autofluorescent signal from single cells from all three independent experiments. Significance was measured using student’s *t*-test (** *p* < 0.01, (*** *p* < 0.001), Figure S4: Graphs of stage-specific, histotype-specific, and lymph node-metastasis-specific mRNA levels of IDS in breast cancer patients from TCGA presented in its graphical user interface UALCAN, Figure S5: Confocal micrographs of MCF-7 cells with no primary antibody (negative control) grown on Type 1 collagen scaffolds and stained for F-actin and DNA, Scale = 20 μm, Figure S6: Graph showing median elastic modulus of Type 1 collagen measured by atomic force microscopy and its interquartile distribution, when polymerized without and in the presence of 50 µg/mL dermatan sulfate (DS).
