# Supplementary material for: Iduronate-2-Sulfatase-Regulated Dermatan Sulfate Levels Potentiate the Invasion of Breast Cancer Epithelia through Collagen Matrix
Source: J Clin Med. 2019 Sep 30;8(10):1562. doi: 10.3390/jcm8101562 (PMC6832158; doi:10.3390/jcm8101562)
Supplement: Supplementary file 1 [file jcm-08-01562-s001.pdf]

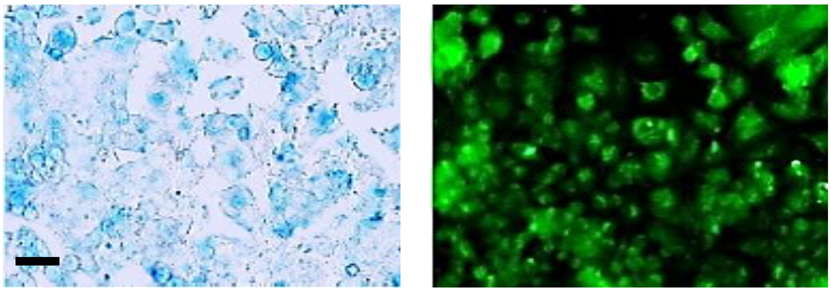

Figure S1. Bright field (left) and laser confocal (right) micrographs of the same field of Alcian blue-stained MCF-7 cells cultured on top of 1 mg/mL Type 1 collagen gels. Images show that the levels of green autofluorescence signals correspond to the intensity of Alcian blue staining in the brightfield. Scale bar = 50 μm.

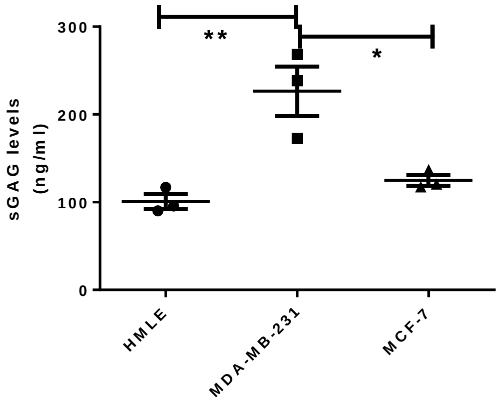

Figure S2. Scatter plot of DMMB assay depicting levels of sulfated glycosaminoglycans (GAGs) in HMLE, MCF-7 and MDA-MB-231 cells grown in 3D Type-I collagen scaffolds. (Data is mean ± SE of three independent experiments). Significance was measured using one-way ANOVA (* $p < 0.05$, ** $p < 0.01$).

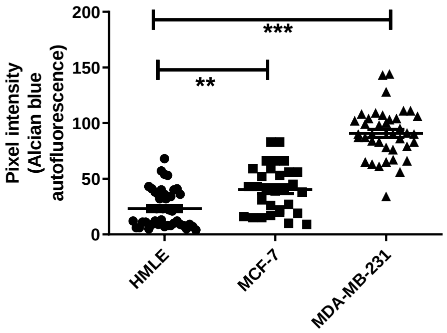

Figure S3. Scatter plot showing pixel intensities of Alcian blue autofluorescence in stained HMLE, MCF-7 and MDA-MB-231 cells cultured on top of 1 mg/mL Type 1 collagen gels. Each spot represents autofluorescent signal from single cells from all three independent experiments. Significance was measured using student's t-test (** $p < 0.01$, (*** $p < 0.001$).

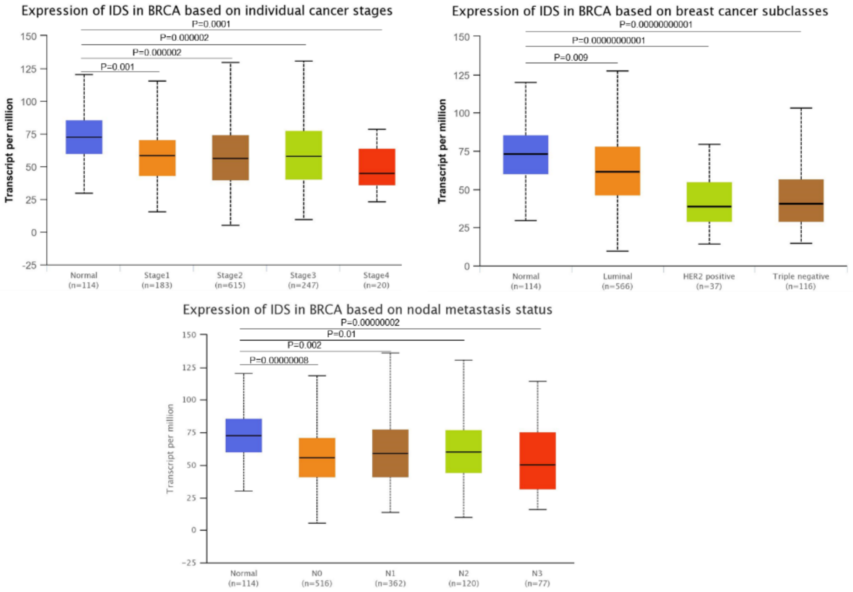

Figure S4. Graphs of stage-specific, histotype-specific, and lymph node-metastasis-specific mRNA levels of IDS in breast cancer patients from TCGA presented in its graphical user interface UALCAN.

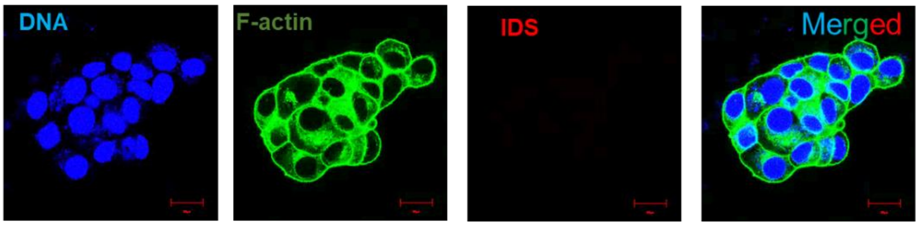

Figure S5. Confocal micrographs of MCF-7 cells with no primary antibody (negative control) grown on Type 1 collagen scaffolds and stained for F-actin and DNA, Scale = 20 μm.

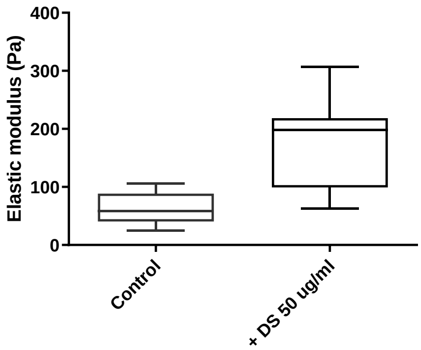

Figure S6. Graph showing median elastic modulus of Type 1 collagen measured by atomic force microscopy and its interquartile distribution, when polymerized without and in the presence of 50 μg/mL dermatan sulfate (DS).
